# A novel seed treatment-based multiplication approach for cassava planting material

**DOI:** 10.1371/journal.pone.0229943

**Published:** 2020-03-06

**Authors:** Eder Jorge de Oliveira, Saulo Alves Santos de Oliveira, Caroline Otto, Titus Alicai, Juan Paulo Xavier de Freitas, Diego Fernando Marmolejo Cortes, Anthony Pariyo, Charles Liri, Gerald Adiga, Andrea Balmer, Dominik Klauser, Mike Robinson

**Affiliations:** 1 Embrapa Mandioca e Fruticultura, Cruz das Almas, Bahia, Brazil; 2 Syngenta Foundation for Sustainable Agriculture, Basel, Switzerland; 3 National Crops Resources Research Institute (NaCRRI), Kampala, Uganda; Banaras Hindu University, INDIA

## Abstract

Cassava (*Manihot esculenta* Crantz) is an important food security crop in many parts of the developing world. The crop’s high yield potential and multitude of uses–both for nutrition and processing–render cassava a promising driver for the development of rural value chains. It is traditionally propagated from stem cuttings of up to 30 cm in length, giving a multiplication rate as low as 1:10. Propagating cassava traditionally is very inefficient, which leads to challenges in the production and distribution of quality planting material and improved cultivars, greatly limiting the impact of investments in crop breeding. The work described in the present study aimed to develop a seed treatment approach to facilitate the use of shorter seed pieces, increasing the multiplication rate of cassava and thus making the crop’s seed systems more efficient. After several tests, formulation was identified, consisting of thiamethoxam 21 g ha^-1^, mefenoxam 1.0 g ha^-1^, fludioxonil 1.3 g ha^-1^, thiabendazole 7.5 g ha^-1^ and Latex 2% as a binder. Plant growing from seed pieces treated with this formulation displayed increased crop establishment and early crop vigor, leading to an improved productivity throughout a full growing cycle. This allowed to reduce the cassava seed piece size to 8 cm with no negative effects on germination and crop establishment, leading to yields comparable to those from untreated 16 cm pieces. This, in turn, will allow to increase the multiplication ratio of cassava by a factor of up to 3. Notably, this was possible under regular field conditions and independently of any specialised treatment facilities. Compared with existing seed production protocols, the increased multiplication rates allowed for efficiency gains of between 1 to 1.9 years compared to conventional five-year cycles. We believe that the technology described here holds considerable promise for developing more reliable and remunerative delivery channels for quality cassava planting material and improved genetics.

## Introduction

Clonally propagated root and tuber crops, such as cassava, sweet potato and yam represent an important component of tropical agriculture. Especially for smallholder farmers, they play a key role as staple and cash crops because of their resilience to abiotic stress, ease of cultivation and flexibility of harvest timing [1, 2, 3]. Additionally, vegetatively propagated crops often allow for a comparatively high yield of readily available food sources [4].

However, low multiplication rates and the perishable nature of planting material make its production and distribution costly and inefficient. For instance, cassava is traditionally propagated through using stem cuttings (hereinafter seed pieces) with 6 to 10 nodes and of up to 30 cm in length [5]. The multiplication ratio between mother and offspring plants is correspondingly low, about 1:5 to 1:10. Moreover, the bulkiness of the planting material presents logistical challenges in the distribution of seed pieces. Consequently, cassava has not benefited from private or public investments in the development and distribution of new cultivars. Farmers mostly rely on informal seed systems, which have few entry points for phytosanitary quality checks or improved genetics [6, 7].

This results in decreased yields and poor quality of the harvested tuberous roots [8, 9]. In South America, for instance, the lack of quality control in the production and dissemination of cassava planting material led to the build-up of disease pressure from a variety of pathogens. These include cassava common mosaic virus, cassava frogskin disease and cassava root rot disease [10]. In East Africa, cassava mosaic disease, cassava brown streak disease and cassava bacterial blight are a major cause of seed degeneration and yield loss [11, 12, 13, 14]. The presence of these pests and diseases leads to average yields of around 8.3–11.2 tons per hectare in East Africa, which is roughly a third of what is believed to be the crop’s potential in this region [15].

Evidence from a range of crops shows that improvement is possible. For instance, the distribution of tolerant varieties and planting material with phytosanitary quality attributes can significantly improve productivity [12]. However, the low multiplication rates of clonally propagated crops have so far limited the economic viability of such interventions [16].

A potential way to address the challenges is to treat planting material with appropriate formulations of protective and/or growth-stimulating compounds. Such formulations can greatly improve a crop’s resilience and productivity [6]. Protective compounds, such as fungicides or insecticides, prevent damage by pests and diseases. Furthermore, certain chemical insecticides have been shown to promote early plant vigour when applied to the seed [17]. This potentially allows for growth and resilience benefits in addition to protecting the crop from biotic stress.

The work described here follows the above hypothesis. To improve production efficiency, we aimed to develop a seed treatment formulation that will allow the use of shorter seed pieces without negatively affecting crop germination and early vigour under field conditions. Multipliers would, therefore, be able to increase the number of seed pieces obtained per parent plant by a factor of up to 3.

Based on a generalised seed system with five annual multiplication cycles and a duration of one year per cycle, we propose a mathematical model to estimate the production of cassava planting material. This allows estimation of the production quantity per period and cutting size. Overall, our proposed hypothesis is that this approach can lead to a much faster multiplication of cassava planting material and thus significantly reduce production costs.

## Materials & methods

### Planting material

The selection of cassava planting material was based on the following criteria: i) vigorous and healthy plants without any signs of insect or disease damage; ii) mature plants aged 10–12 months with fully grown tuberous roots; iii) tissue from the base, middle and top part of the stem, providing they were mature enough to generate vigorous plants; iv) the diameter of the pith was at least 50% of the cutting diameter. After selecting the plants, the stems were harvested and then cut using an adapted electric rotary saw with a sharp blade to avoid splintering.

Four widely cultivated cassava varieties (‘BRS Formosa’, ‘Cascuda’, ‘Fécula Branca’ and ‘IAC 90’) were used in the experiments in Brazil. Two additional, widely-used, virus-tolerant varieties (‘NASE19’ and ‘NAROCASS 1’) were used in Uganda (main traits listed in Table 1).

**Table 1:** Characteristics of cassava varieties used for the validation of the seed treatment technology under field conditions.

Variety	Plant height [m]	Fresh root yield [t ha^-1^]	Starch content [t ha^-1^]	Dry matter content [%]	Other important traits
‘BRS Formosa’^1^	1.80 ± 0.22	29.72 ± 5.25	8.99 ± 1.6	35.00 ± 1.26	Resistant to CBB
‘Cascuda’^2^	1.85 ± 0.26	27.77 ± 3.32	8.23 ± 1.19	34.23 ± 1.08	Large adaptability
‘Fécula Branca’^2^	1.72 ± 0.25	32.16 ± 9.56	8.48 ± 0.97	34.87 ± 4.35	High root yield
‘IAC 90’^1^	1.47 ± 0.30	14.32 ± 6.22	4.04 ± 1.82	33.30 ± 1.94	High root yield
‘NASE 19’	1.60 ± 0.20	35.00 ± 10.00	N/A	31.00 ± 6.00	Resistant to CMD, Tolerant to CBSD, High root yield
‘NAROCASS 1’	1.60 ± 0.20	35.00 ± 10.00	N/A	31.00 ± 6.00	Resistant to CMD, Tolerant to CBSD, High root yield

Source:

^1^[18]

^2^[19, 20, 21, 22]

CBB (cassava bacterial blight); CMD (cassava mosaic virus); CBSD (cassava brown streak disease)

### Assessment of seed pieces

**Fig 1:**
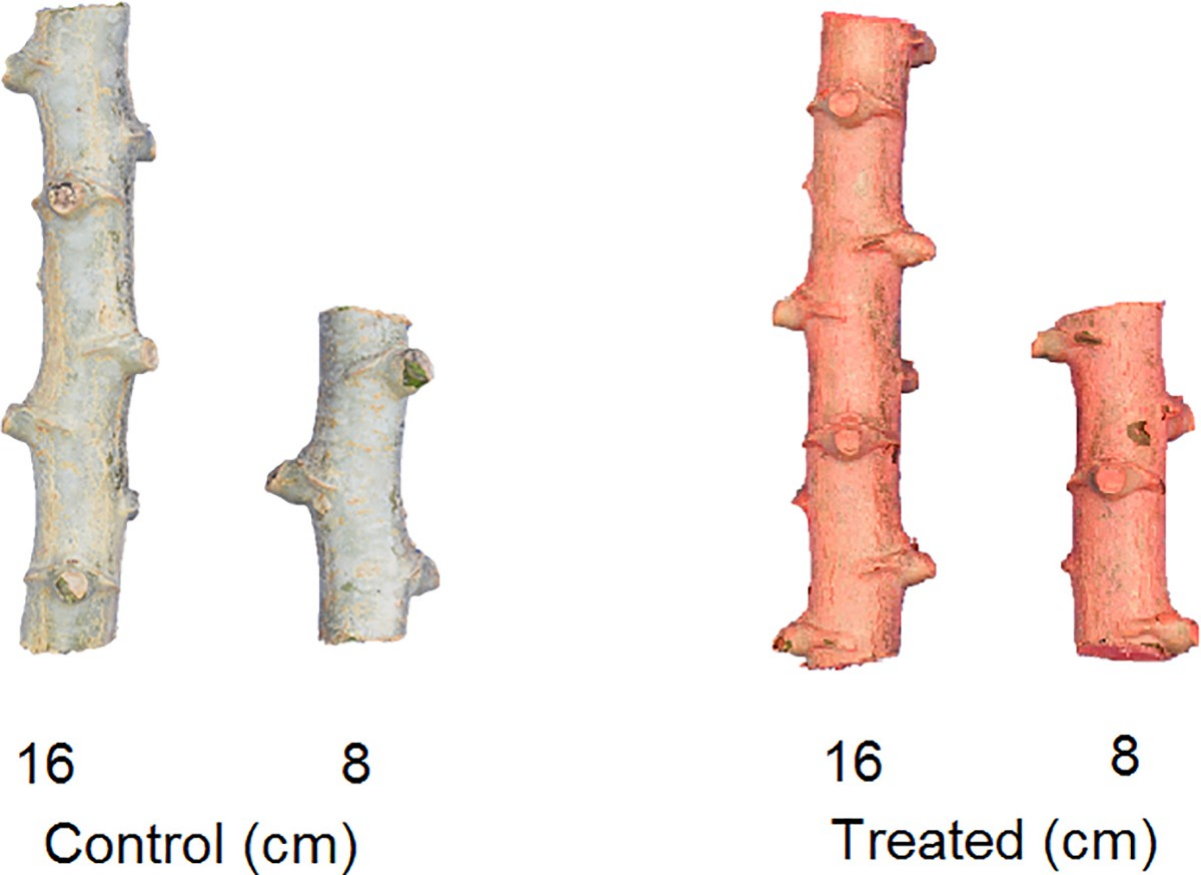
Depiction of cassava seed pieces (16 and 8 cm), untreated (left) and treated (right) with the seed treatment formulation, as used in this work.

Stem cutting sizes used in the field and growth chamber trials were 8 and 16 cm (Fig 1). Planting material was selected based on the criteria described previously for the experiments conducted in Brazil. The 16 cm version was used as a control for the different treatments because most Brazilian farmers traditionally use this size. The number of buds on the 8 cm cutting size ranged from 2–4, 83% had two buds and 17% had more than two buds. Buds on the 16 cm seed pieces ranged from 3–7, 31% had three buds, 57% had 4–5 buds, and 12% had more than five buds. On the other hand, cutting sizes of 12 cm for short seed pieces and 24 cm for control were used for the experiments carried on in Uganda because 24 cm length is the local recommendations to farmers for selecting cassava stem cuttings. The control size was selected based on predominant local cassava cultivation methods.

### Trial locations

All trials and experiments were conducted in one or several of the locations listed in Table 2.

**Table 2:** Locations of field trials including institution names, coordinates and altitude.

Institution	Location	Coordinates	Altitude (m asl)
Bahiamido S.A. (Bahiamido)	Laje, Bahia, Brazil	13°10’56” S; 39°25’30” W	220
Universidade Federal do Recôncavo da Bahia (UFRB)	Cruz das Almas, Bahia, Brazil	12°40’39’ S; 39°06'23’ W	235
Embrapa Mandioca e Fruticultura	Cruz das Almas, Bahia, Brazil	12°39'11’ S; 39°07'19’ W	226
Namulonge	Namulonge, Wakiso, Uganda	00°31'30’ N; 32°36'54’ E	1150
Kigumba	Kigumba, Kiryandongo, Uganda	01°81'28’ N; 32°01'27’ N	1072

### Seed treatment application

The dosage of the agrochemicals was calculated according to the volume of absorption of the seed pieces before the treatment. An initial cassava sample with each of the different cutting sizes was dipped in water for three minutes to identify the volume absorbed by seed pieces during this period. Then, the volume of absorption in one hectare was calculated (number of seed pieces per ha x absorption per stake) and use to estimate the total volume of product required (TVPR):

$TVPR=\frac{dosage\;of\;product*slurry\;required}{absorption\;volume*{ha}^{-1}}$. In addition to the agrochemicals, a binding agent (latex, 2%) was used to increase treatment adhesion to the seed piece surface. All agrochemicals were applied as a slurry seeking to provide uniform coverage of the seed pieces. The slurry was prepared according to the following sequence: i) water; ii) latex; iii) fungicides; iv) insecticide. The pH value was adjusted between 6.5 to 7.0 using HCl to avoid hydrolysis of some chemicals. After treatment, the seed pieces were left to dry for 8 hours at room temperature and then placed in high-density polyethylene (HDPE) Raschel mesh bags (used for onions and potatoes).

### Selection of treatment formulations

The experimental setup for testing and validating treatment formulations followed a completely randomized in factorial scheme: dosage × type of agrochemicals, with three replicates consisted of ten seed pieces. After the seed treatment, the pieces were planted directly in growth chambers (sand bed that are covered with plastic blanket, forming a high humidity chamber), containing washed sand and vermiculite (3:1 ratio, respectively) as the substrate for growth. The seed pieces were growth at 28/20°C day/night temperature, and 70 to 80% relative humidity (irrigated daily by automated microaspersion). Light conditions were natural light. Thirty days after planting (DAP), the following parameters were assessed for selection of treatment: 1) Phytotoxicity: expressed as percentage of affected plants and determined by the presence of chlorosis or yellowing of leaves; 2) Plant height: expressed in cm, measured from the soil base to the insertion of the youngest leaf; 3) Shoot dry weight: expressed in g considering the weight of all leaves, petioles and stems, after drying in an oven for four days at 60°C; 4) Root dry weight: expressed in g and obtained after drying in an oven for four days at 60° C. The list of seed treatment formulations and concentrations is featured in S1 and S2 Figs and discussed in the results section.

For statistical analysis of trials, the traits were assessed using a linear model. The linear model is shown below (model 1):

$$Y_{ijk}=\mu +A_{i}+D_{j}+{AD}_{ij}+T_{l}+\varepsilon ijk$$

Whereas *Y*_*ijk*_ is the variable to be analysed; μ is the general mean; *A*_*i*_ is the main effect of the *i-th* Agrochemical (fixed); *D*_*j*_ is the main effect of the *j-th* dosage (fixed); *AD*_*ij*_ is the interaction effect of the *i-th* agrochemical and the *j-th* dosage (fixed); *T*_*l*_ is the contrast between the additional treatment and double factorial; *εijk* is the experimental error, assumed independent ~N (0, σ^2^). Additional treatment refers to controls (Unt1 = cassava seeds treated only with water; Unt2 = cassava seeds treated only with latex, 2%). A two-way analysis of variance (ANOVA) model, was used to assess the effects of dosage × type of agrochemicals and their interaction upon the growth of cassava plants. The means were separated using the least significant differences (LSD) test with significance set at *p* < 0.05. The statistical analyses were performed in R v.3.3.3 [27].

### Phytopathology tests

Treated and untreated seed pieces were planted in plastic cups (200 mL), filled with 20g of vermiculite, 5 g of the inoculum and 20 mL of sterile water. The inoculum was produced in a sand-cornmeal substrate (ratio 3:1), which was infested with different pathogen isolates (*Fusarium* spp., *Lasiodiplodia* spp., and *Phytophthora* spp.). A second 200 mL plastic cup was used to cover the seed pieces to maintain moisture. The evaluation was performed 30 days after planting for the following parameters: i) rooting score (0 = no roots or callus formation; 1 = presence of callus and/or initial roots; 2 = presence of few developed roots; 3 = presence of roots); ii) incidence of fungal colonization (0 = no fungal colonization; 1 = fungal colonization of < 1/3 of cutting length; 2 = fungal colonization > 1/3 and < 2/3 of cutting length; 3 = fungal colonization > 2/3 of cutting length).

The experiment was set in a complete randomized design with three replications of 20 seed pieces per plot. Statistical analyses were done using the model 1 previously described. The analysis of variance (ANOVA) assumptions were tested, and the LSD test was adopted for means comparison of the treatment (*p* < 0.05). The statistical analyses were performed in R v.3.3.3 [27].

### Field experiments

The impact of treatment on early plant vigour under field conditions was analysed based on the most suitable seed treatment formulation obtained from the preliminary experiments (Table 3). Here, 8 and 16 cm seed pieces from the cassava varieties ‘BRS Formosa’ and ‘IAC-90’ were treated with the selected formulation of agrochemicals or planted without treatment as a control. These varieties were chosen based on the availability of sufficient propagation material. Land preparation consisted of one disc-ploughing pass followed by two three-disc harrowing passes and one-furrow opening using conventional cassava planting machines. Two to three DAP, a pre-emergence herbicide was applied (flumioxazin 80 g ha^-1^); 50 to 60 DAP; weed control took the form of one manual hoeing. The seed pieces were placed horizontally at an approximate depth of 0.10 m in the furrows, and then manually covered with soil, using a hoe. The cultivation was performed according to the recommendations of Souza et al. [23]. The seed pieces were planted at a spacing of 0.8 m × 0.8 m. The plots consisted of four rows with five plants in a randomised complete block design, with four replicates.

**Table 3:** Composition of seed treatment formulations used in Brazil and Uganda.

Active ingredient	Compound class	Reference (g ha^-1^)	Brazil (g ha^-1^)^a^	Uganda (g ha^-1^)
Thiamethoxam	Insecticide	420.0	21.0	21.0
Mefenoxam (Metalaxyl-M)	Fungicide	45.0	1.0	1.7
Fludioxonil	Fungicide	15.0	1.3	4.2
Azoxystrobin	Fungicide	90.0	-	-
Thiabendazole	Fungicide	62.0	7.5	-
Latex	Binding Agent	-	2.0%	-
Vinyl Silk White	Binding Agent	-	-	2.0%

Sixty DAP, an unmanned aerial vehicle (Phantom 3 Pro, DJI Products) took pictures in RGB colour system. Processing used an algorithm based on artificial neural networks of the MultiLayer Perceptron type to study soil cover through pattern recognition. The algorithm was implemented via SisCob software [24]. Vegetative coverage for each plot was expressed as the percentage of ground surface covered by the cassava plants from the different treatments.

The agronomic performance was measured by evaluating the germination of each plot at forty-five DAP and the following parameters at twelve months after planting:

1) Plant height, measured in m; 2) Plant stand, measured by counting the number of plants per plot and then expressing this as a percentage of the expected number of plants per plot; 3) Above-ground biomass i.e., stems, petioles and leaves, in t ha-1; 4) Fresh root yield, in t ha-1; and 5) Dry yield, in t ha^-1^ was derived as a product of fresh root yield and dry matter content, expressed in percentages, measured by specific gravimetric analysis according to [25].

For statistical analysis of unbalanced trials, the traits were assessed using a linear mixed model approach for a randomised completely block design, modelling environment (*E*), block and error as random effects and cassava varieties (*V*), treatments of seed pieces (*T*) and cutting sizes (*C*) as fixed effects. The linear mixed model was:

$$Y_{ijkl}=m+E_{i}+V_{j}+T_{k}+C_{l}+{EV}_{ij}+{ET}_{ik}+{EC}_{il}+{VT}_{jk}+{VC}_{jl}+{TC}_{kl}+{EVT}_{ijk}+{EVC}_{ijl}{+ETC}_{ikl}+{VTC}_{jkl}+{EVTC}_{ijkl}$$

Whereas *Y*_*ijkl*_ is the trial for 4-factorial combination of the *i-th* environment (*i* = 1,2,3), the *j-th* variety; the *k-th* treatment and the *l-th* cutting size; *m* is the general mean; *E*_*i*_ is the random main effect of the *i-th* environment; *V*_*j*_ is the the fixed main effect of the *j-th* variety; *T*_*k*_ is the fixed main effect of the *k-th* treatment; *C*_*l*_ is the fixed main effect of the *l-th* cutting size; *EV*_*ij*_ is the random interaction effect of the *i-th* environment and the *j-th* variety; *ET*_*ik*_ is the random interaction effect of the *i-th* environment and the *k-th* treatment; *EC*_*il*_ is the random interaction effect of the *i-th* environment and the *l-th* cutting size; *VT*_*jk*_ is the fixed interaction effect of the *j-th* variety and the *k-th* treatment; *VC*_*jl*_ is the fixed interaction effect of the *j-th* variety and the *l-th* cutting size; *TC*_*kl*_ is the fixed interaction effect of the *k-th* treatment and the *l-th* cutting size; *EVT*_*ijk*_ is the random interaction effect of the *i-th* environment, *j-th* variety and the *k-th* treatment; *EVC*_*ijl*_ is the random interaction effect of the *i-th* environment, *j-th* variety and the *l-th* cutting size; *ETC*_*ikl*_ is the random interaction effect of the *i-th* environment, *k-th* treatment and the *l-th* cutting size; *VTC*_*jkl*_ is the fixed interaction effect of the *j-th* variety, *k-th* treatment and the *l-th* cutting size; *EVTC*_*ijkl*_ is a random residual comprising both the interaction effect of the *i-th* environment, the *j-th* variety, the *k-th* treatment, the *l-th* cutting size, and the error term associated with a mean *Y*_*ijkl*_, assumed independent ~N (0, *σ*^2^).

For field trials in Uganda, disease-free cassava stems were obtained from cassava pre-basic seed fields in Kigumba. Stem lengths of 8cm, 12cm, 16cm and 24cm for the varieties ‘NASE 19’ and ‘NAROCASS 1’ were cut using rotary cutter. The cut stakes were packed into polypropylene bags and dipped into 10-liter plastic buckets of different plant protection products for three minutes. The stakes were drained by hanging the bags on a draining table and seed pieces were left to dry at ambient temperature.

The experiments were planted in split-split plot randomised design replicated four times. The main plots treatments were the plant protection products, sub-plot treatments were varieties and sub-sub plot treatments were the cutting lengths. 81 plants were planted per plot and a spacing of 0.9 m between rows and 0.8 m between plants was used. Inter-plot, inter-treatment and inter replication spacings between plots were 1.6m and 1.8 m, respectively.

For statistical analysis of Uganda field trials, we used a linear mixed model based on the split-split plot designs with a factorial experiment involving two varieties, two cutting sizes and two treatments of seed pieces tested at the two environments. The linear mixed model was:

$$Y_{ijklm}=m+E_{i}+T_{j}+{ET}_{ij}+{\delta ijm+V}_{k}+{EV}_{ik}+{TV}_{jk}+{ETV}_{ijk}+\omega ijkm+C_{l}{+EC}_{il}+{TC}_{jl}+{VC}_{kl}+{ETC}_{ijl}{+EVC}_{ikl}+{TVC}_{jkl}+{ETVC}_{ijkl}+\varepsilon ijklm$$

whereas *Y*_*ijklm*_ is the variable to be analyzed; m is the general mean; *E*_*i*_ is the effect of the *i-th* Environment (random); *T*_*j*_ is the effect the *j-th* treatment (fixed); *ET*_*ij*_ is the effect of the interaction of the *i-th* environment with *j-th* treatment (random); *δijm* is first restriction error (pooled error a), assumed independent ~N (0, $\sigma_{T}^{2}$); *V*_*k*_ is the effect of the *k-th* variety (fixed); *EV*_*ik*_ is the effect of the interaction of the *i-th* environment with the *k-th* variety (random); *TV*_*jk*_ is the effect of the interaction of the *j-th* treatment with the *k-th* variety (fixed); *ETV*_*ijk*_ is the effect of the interaction of the *j-th* treatment in the *i-th* environment with the *k-th* variety (random); *ωijkm* is the second restriction error (pooled error b), assumed independent ~N (0, $\sigma_{V}^{2}$); *C*_*l*_ is the effect of the *l-th* cutting size (fixed); *EC*_*il*_ = effect of the interaction of the *i-th* environment with the *l-th* cutting size (random); *TC*_*jk*_ = effect of the interaction of *the j-th* treatment with the *l-th* cutting size (fixed); *VC*_*kl*_ = effect of the interaction of the *k-th* variety with the *l-th* cutting size (fixed); *ETC*_*ijl*_ = effect of the interaction of the *j-th* treatment in the *i-th* environment with the *l-th* cutting size (random); *EVC*_*ikl*_ = effect of the interaction of the *k-th* variety in the *i-th* environment with the *l-th* cutting size (random); *TVC*_*jkl*_ = effect of the interaction of the *j-th* treatment with *k-th* variety with the *l-th* cutting size (fixed); *ε*_(*ijkl*)*m*_ = within error blocks within treatments, varieties and cuttings size in environments (error c), assumed independent ~N (0, *σ*^2^).

For both counties, the significance of fixed effects was tested by Wald-type F-statistics, and the variance components for random effects were estimated using the restricted maximum likelihood (REML) method. The odds ratio test was used to evaluate the significance of random effects. The function *lsmeans* was used to calculate marginal mean differences between groups and evaluate significantly mean differences with Tukey HSD adjustment to *p*-values, when a significant main effect or an interaction was observed. The functions *lmer* and *cld* within the lmer Test package [26] in R v.3.3.3 [27] were used to assess the effect of the treatments and their interactions, and a compact letter display of the *lsmeans* was used for plotting.

### Seed system modelling and calculations

The mathematical model used for calculations to predict seed piece quantities after a certain amount of time and to further compare the differences between systems with longer or shorter seed pieces was established based on multiplication factors and cycles obtained from Brazil (Embrapa, personal communication). For the calculations, the model was implemented in R Studio v. 1.1.456 [27]. The model is shown in equations 1–3.

- Equation 1: $Q(x)={(A}_{n}*\frac{\left(x-(n-1)*365\right)}{365}+\sum_{j=0}^{n-1}A_{j})*i$, whereas Q(x) is the amount of seed pieces in the multiplication system after x days. *n* is given by:
- Equation 2: $n=\sum_{k=0}^{5}H_{k}((x-1)-k*365)$, *H(x)* describes the Heaviside function as following: $H(x):R\to \{0,1\},x\mapsto \left\{ \begin{array}{c} 0:x<0\\ 1:x\geq 0 \end{array}\right.$
- *A*_*n*_ = {*A*_0_,*A*_1_,*A*_2_,*A*_3_,*A*_4_,*A*_5_}, is based on
- Equation 3: $A_{n}=(\prod_{k=1}^{n-1}\left(A_{k}\right))*(f_{n}-1),n\in [2,\infty [,{whereas\;A_{0}=0\;and\;A}_{1}=f_{1}$, values for multiplication factors *f*_*n*_ are listed below. Inputs for Q(x) are *A*_*n*_ (number of seed pieces generated per year *n*, based on multiplication factors), *n*, based on days x and *i*, initial amount of tissue culture plantlets.

For 24 cm seed pieces: *f*_*n*(24*cm*)_ = {0,9,10.6,10.6,5.3,5.3};*therefore A*_*n*_ = {0,9,86.4,915.8,4348.3,23′046.2}.

For 16 cm seed pieces: *f*_*n*(16*cm*)_ = {0,9,16,16,8,8};*therefore A*_*n*_ = {0,9,135,2160,16′128,129′024}.

For 8 cm seed pieces: *f*_*n*(8*cm*)_ = {0,9,32,32,16,16}; *therefore A*_*n*_ = {0,9,279,8928,138′240,2′211′840}. Multiplication factors (*f*_*n*_) are based on the following assumptions: i) Current traditional approaches are based on a multiplication ratio of 1:8 per year; ii) The initial tissue culture and greenhouse multiplication steps (year one) are based on the most conservative approach.

## Results

The initial selection of formulations was based on existing treatment formulations for clonally propagated crops, using thiamethoxam 420 g ha^-1^, mefenoxam 45 g ha^-1^, fludioxonil 15 g ha^-1^, azoxystrobin 90 g ha^-1^, and thiabendazole 62 g ha^-1^. However, as planting densities for crops treated with this formulation were up to ten times higher than the ones for cassava, we started with a dilution factor of 5% and 10% to avoid potential phytotoxic effects. Indeed, the treatment with mefenoxam + fludioxonil + azoxystrobin, exhibited high phytotoxicity rates (83.33 and 96.67%, respectively) compared to controls. Thiamethoxam at 10% also caused phytotoxicity on 33.0% of the plants, although the phytotoxicity symptoms of the thiamethoxam were much less prominent than those caused by the combination of different fungicides (S1 Fig).

As the combined application of mefenoxam + fludioxonil + azoxystrobin was causing phytotoxicity in cassava plants, regardless of application rates, other alternative fungicides were tested to verify the effects on cassava growth. New formulations were evaluated, composed of mefenoxam + fludioxonil + thiabendazole and mefenoxam + fludioxonil at 1.1%, 2.2% and 4.4% dilution factor (S2 Fig).

The plants treated with the different dosages of these agrochemicals did not show any symptoms of phytotoxicity. Regarding the additional agronomic traits, the agrochemical treatments displayed a positive effect on the development of the plants, with significantly higher values for shoot and root dry weight by most of the treatments compared to control (S2 Fig).

The initial tests of cassava seed growth in substrate containing root rot pathogens showed poor rooting of untreated pieces (S3 Fig). In contrast, both treatment mefenoxam + fludioxonil + thiabendazole and mefenoxam + fludioxonil resulted in a high rooting index, especially at dosages of 2.2% and 4.4% (score > 1.89) in comparison with the control (0.50). Besides, treated cassava seeds with both chemical combinations exhibited a low incidence of external and internal fungi colonization compared to untreated seeds.

**Fig 2:**
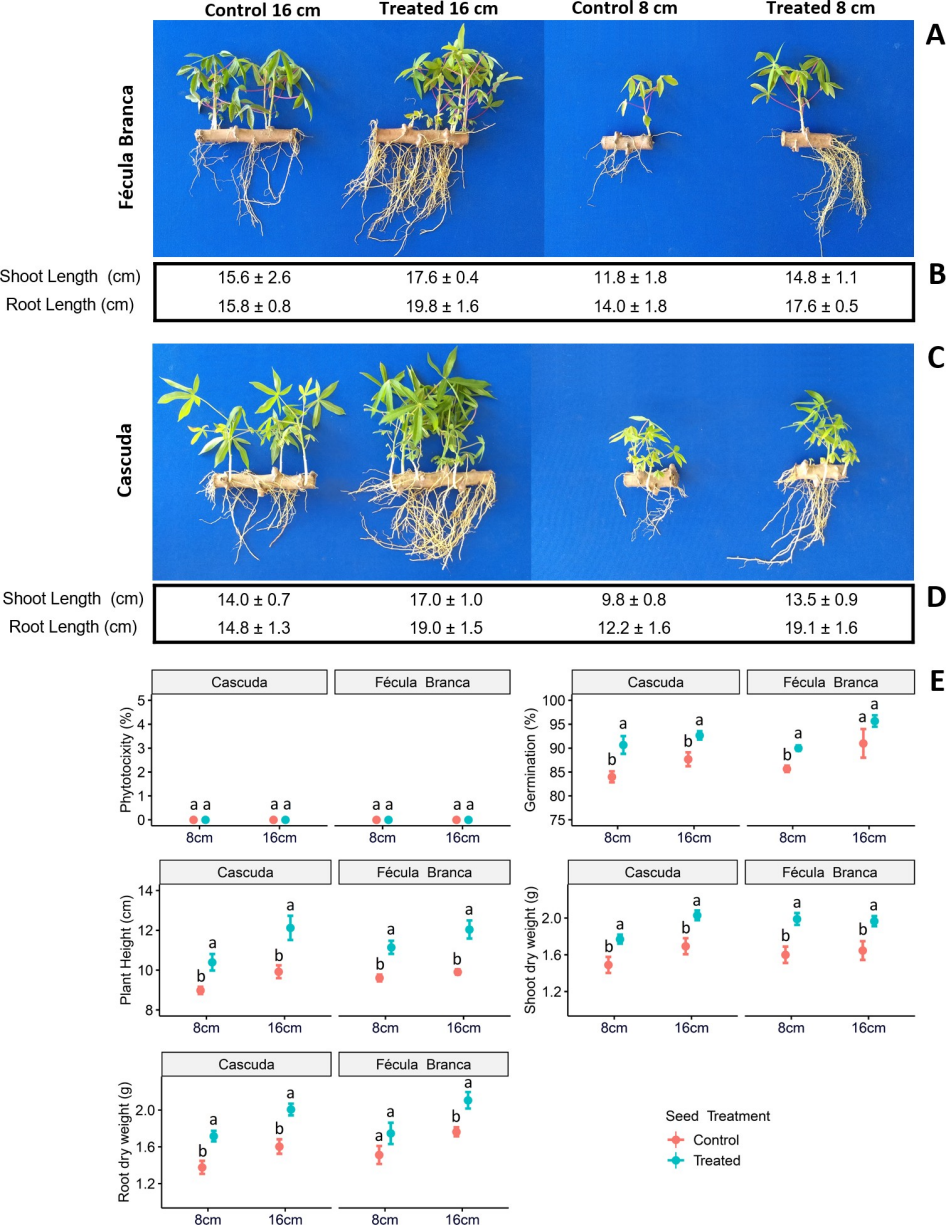
Effect of the seed treatment on plantlets of varieties ‘Cascuda’ and ‘Fécula Branca’, 30 days after planting in growth chambers. In **(A)** and **(C)**, different cutting sizes are shown, 8 and 16cm, both with and without the treatment. Underlying parameters for shoot and root length are listed in **(B)** and **(D)**. Phytotoxicity (%), germination (%), plant height (cm), shoot dry weight (g) and root dry weight (g) are shown in **(E)**. Values displayed represent averages across 3 replicates with 20 samples per plot each. Error bars depict standard errors. Different letters per variety indicate significant differences between cutting sizes, both with and without treatment, *p* < 0.05 by LSD test.

Fig 2 shows plantlets at 30 days after planting, grown in growth chambers, with initial seed piece lengths of 8 (shorter) and 16 (longer) centimeters, comparing treated and untreated seed pieces. The controls (untreated) showed impaired development of plantlets growing from shorter seed pieces (Fig 2A, 2B, 2C and 2D). The plants originating from the 8 cm untreated seed piece were shorter and developed fewer roots and less shoot biomass. In contrast, to the untreated control pieces, the plantlets originating from 8 cm treated seed pieces do not show negative effects on the reduction in roots and shoot length. In comparison to the 16cm control (untreated), the performance of the shorter, treated pieces is equal or better for nearly all of the parameters assessed (Fig 2E): phytotoxicity, germination, plant height, as well as root and shoot dry weight. Moreover, the plantlets from 8 cm treated seed pieces perform similarly or are only inferior to those from treated, longer pieces.

**Fig 3:**
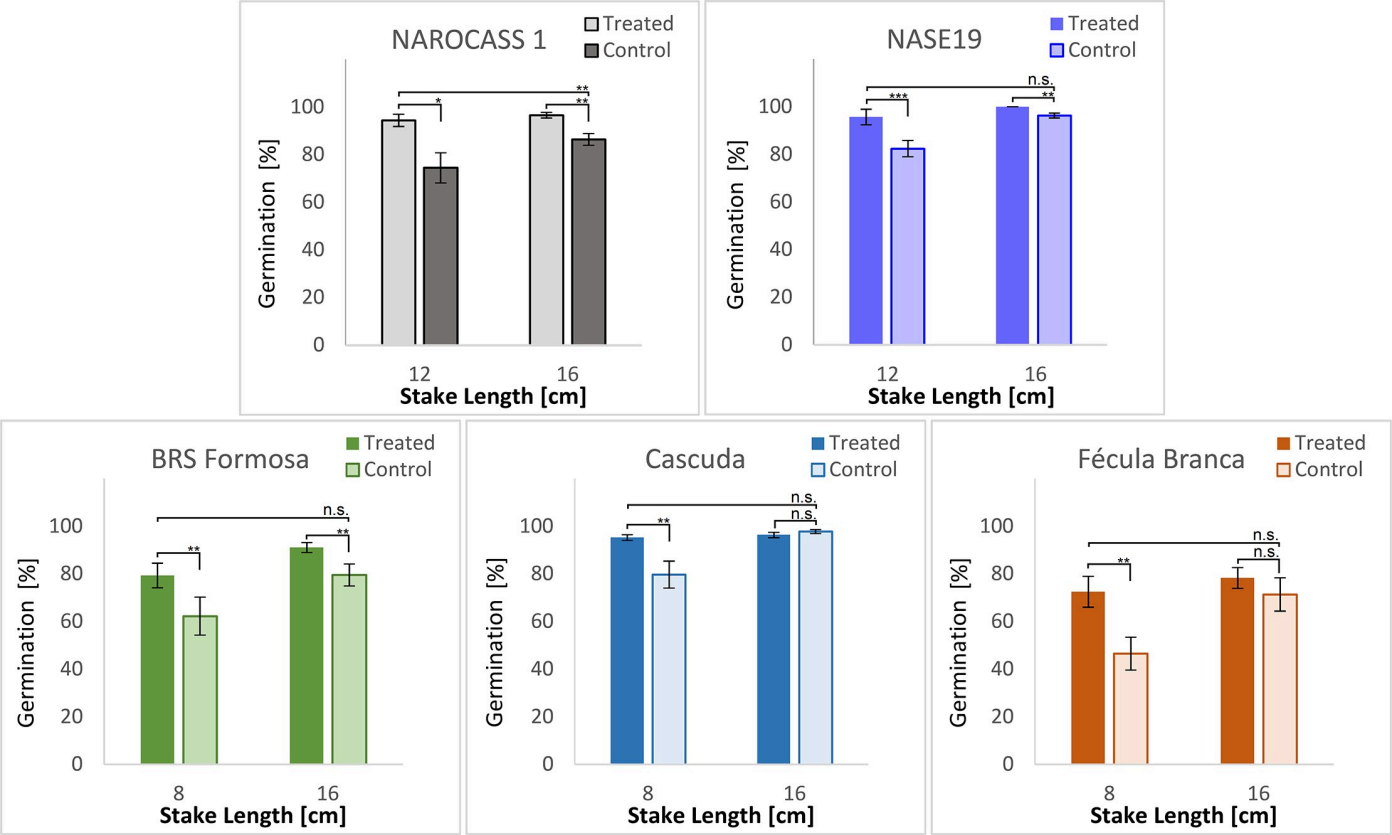
Results from experiments performed in Uganda, using two local varieties (NASE 19’ and ‘NAROCASS 1’) and in Brazil, using three local varieties (‘BRS Formosa’, ‘Cascuda’ and ‘Fécula Branca’). Seed pieces with lengths of 12 and 24 cm (Uganda) and 8 and 16 cm (Brazil) under treated and control conditions were compared. Treatment formulations are explained in Table 3. Germination percentage was assessed 30 days after planting. Values are averages across 4 replicates of 4 plots (with 81 plants per plot) each. Error bars represent standard deviation. Statistical analysis was performed with a t-test, assuming unequal variances. Significance levels depicted are as follows: n.s.: non-significant; *: *p* < 0.05; **: *p* < 0.01; ***: *p* < 0.005.

Based on these first, short-time growth chamber assessments, further experiments were performed to examine whether these tendencies and characteristics could be confirmed over a longer period and under field conditions. Finally, key parameters at harvest, such as shoot and root yield were also measured. Fig 3 shows the germination rates of treated and untreated seed pieces both in Brazil and Uganda. For both countries and independently of varieties, a treatment effect could be observed, with treated seed pieces showing higher germination rates compared to control seed pieces of the same length.

**Fig 4:**
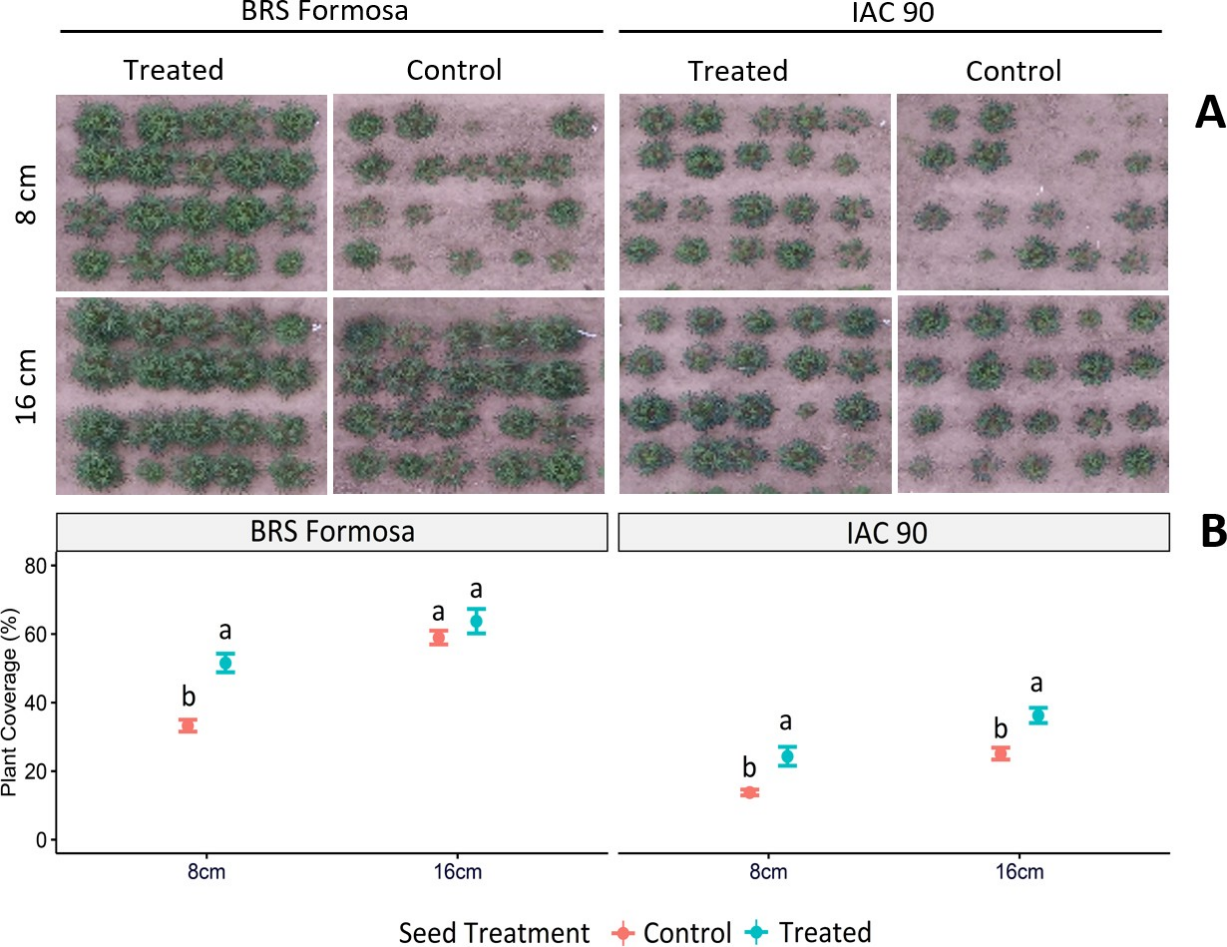
**A)** Plant coverage (%) of varieties ‘BRS Formosa’ and ‘IAC90’, evaluated 60 days after planting under field conditions. Rows are grouped in plots of 8 cm and 16 cm seed piece length, respectively. Columns are depicted correspondingly to applied treatment; treated or control (treated only with water), as marked on the top of each column. In **B)**, the corresponding cover crop percentages are depicted for both of the varieties which have been trialled, as well as for the different lengths and treatments. Values are averages across 4 replicates of 10 samples per plot. Error bars represent standard error. Different letters for each variety indicate significant differences between cutting sizes, both with and without the treatment, *p* < 0.05 by LSD test.

Fig 4 shows plant coverage of the varieties ‘BRS Formosa’ and ‘IAC 90’ 60 DAP. Results indicate that, under field conditions, plants from untreated 8 cm seed pieces do not germinate as well as plants from either treated or longer stems. Plant coverage of treated or longer seed pieces is also clearly higher.

**Fig 5:**
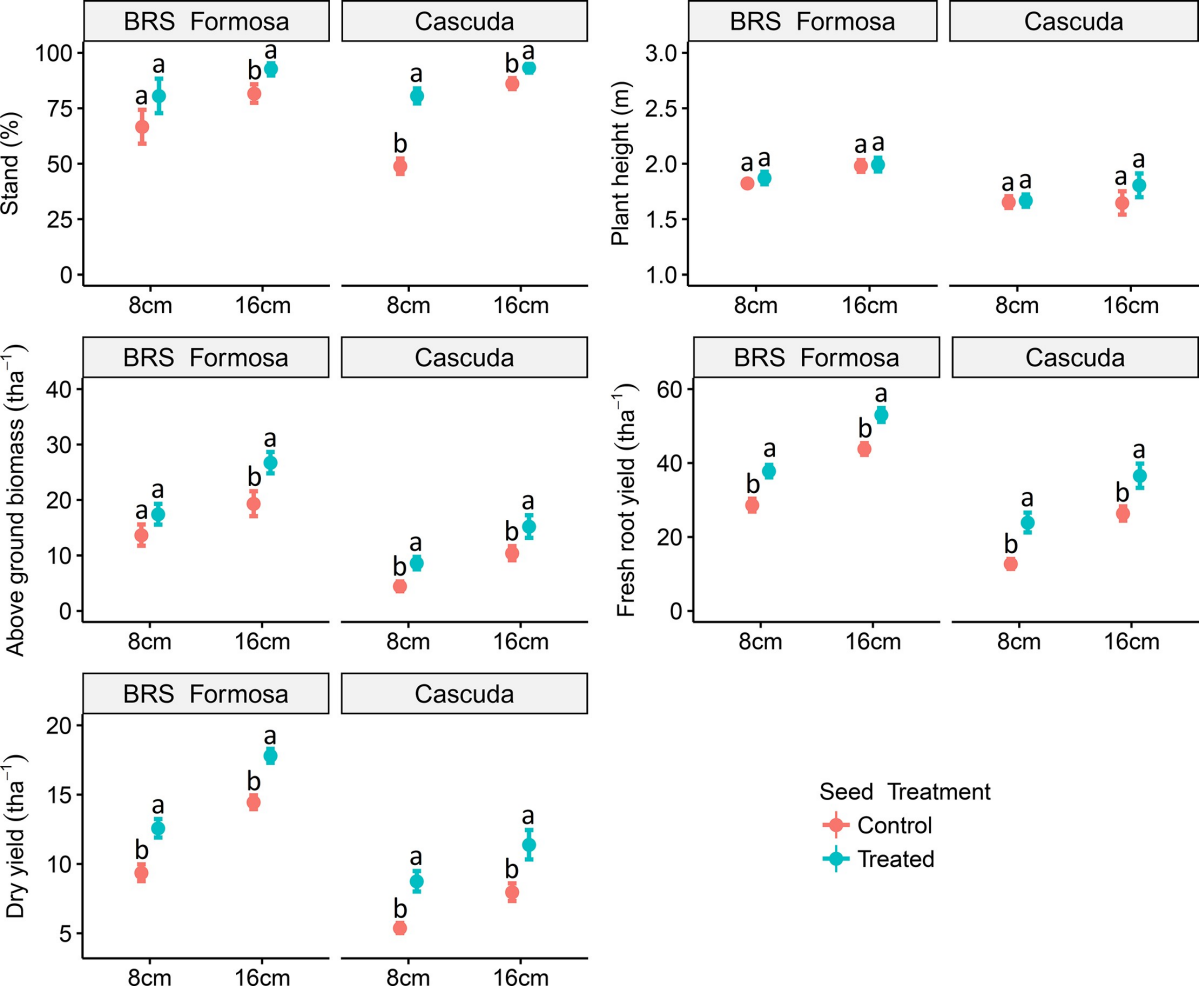
Parameters from the field trials for the varieties ‘BRS Formosa’ and ‘Cascuda’, harvested and evaluated 12 months after planting. Assessed parameters include plant stand (%); plant height (m), above-ground biomass (t ha^-1^) and fresh root yield (t ha^-1^). Treated and untreated seed pieces of lengths 8 and 16 cm are compared. Values are averages across 4 replicates of 20 samples per plot and three environments in Northeast Brazil (Embrapa, UFRB, Bahiamido). Error bars represent standard error. Different letters for each variety indicate significant differences between different cutting sizes, both with and without the treatment, *p* < 0.05 by Tukey Honest Significant Difference test.

Fig 5 summarises parameters measured at harvest (12 months after planting): plant stand, plant height, above-ground biomass, fresh root yield, and dry yield (Fig 5). The numerical results confirm the visual impressions and resulting numbers of the trials depicted in Fig 5A) and 5B): Untreated 8 cm seed pieces display impairment in plant stand. Their results differ significantly from those for treated 8 cm and both treated and untreated 16 cm pieces. Overall, the plants derived from treated stems perform better than untreated of the same size. Fig 5 shows that the differences in plant height are insignificant, regardless of initial seed piece length or treatment. On the other hand, a tendency towards increased biomass production by treated plants was observed, as the treated seed pieces displayed increased biomass production in comparison to their corresponding untreated controls. By harvest time (12 months after planting), the treated seed pieces had produced more root biomass than untreated pieces of the corresponding lengths. In addition, treated 8 cm pieces led to yields comparable to those from untreated 16 cm pieces.

**Fig 6:**
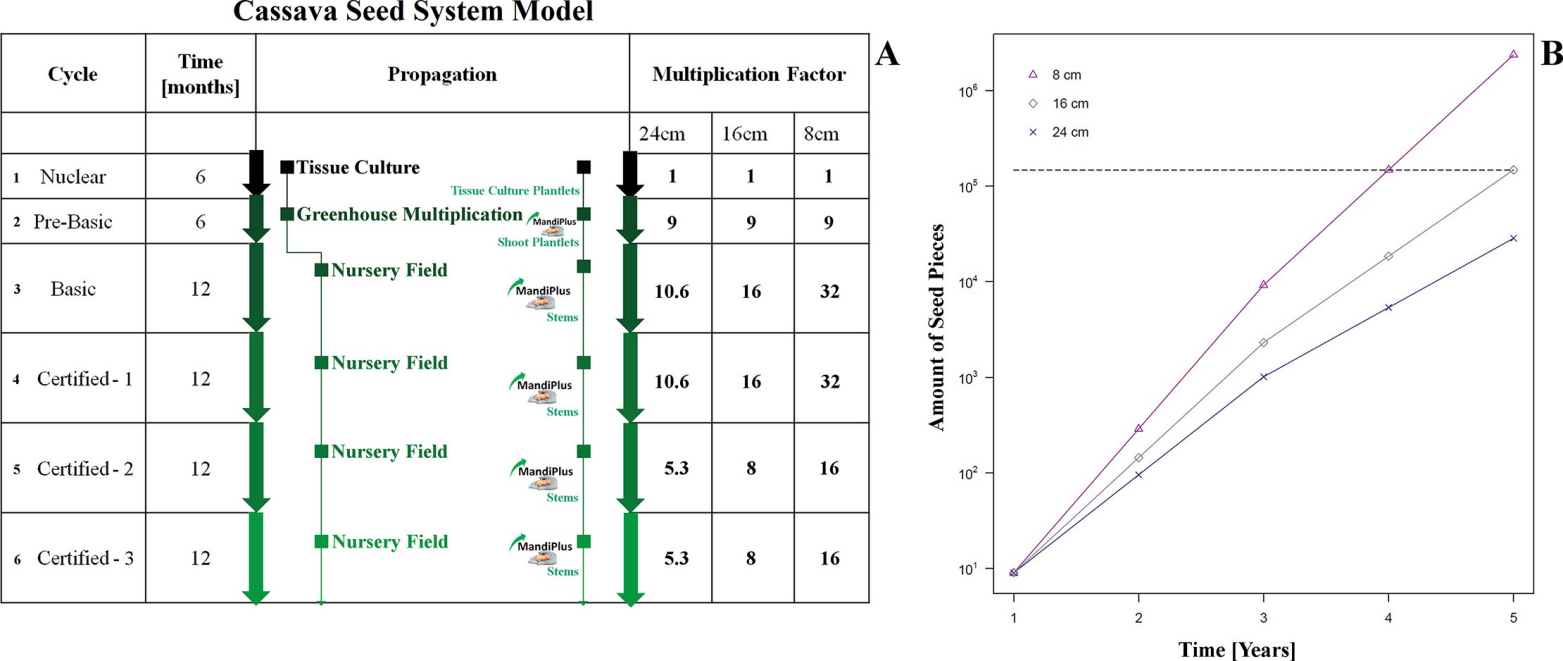
Generalised model of the cassava seed system. In **A)**, a generalised cassava seed system is shown. The initial year in multiplication consists of tissue culture production (Nuclear, 1) and subsequent plantlet multiplication in a greenhouse (Pre-Basic, 2). Further, the basic seed multiplication cycle (Basic, 3) and the certified cycles (Certified, 4–6) are entered, each with the duration of one year. In the basic cycle, shoot plantlets are planted, after one year of basic seed multiplication, cassava seed stems can be used. The resulting multiplication factor, depending on seed piece length is depicted on the right. For the basic and first certified cycles (3 and 4), the multiplication factors can be double the factors of the two last certified cycles (5 and 6), as intensive crop systems associated with the use of irrigation allow two stem harvests per year. A full iteration through the entire seed system equals 5 years. Multiplication factors are indicated per year, based on entering the seed system with one single tissue culture plantlet. Asterisks depict where the treatment technology is recommended for application. From the greenhouse multiplication step onwards, a benefit from the technology’s use is expected. In **B)**, the seed piece production is shown over the full duration of five years. Production for the three different lengths (8, 16, and 24 cm) is depicted. The dashed line shows the equal amount of seed pieces for 8 cm after 4 years and for 16 cm after 5 years, 147’456 seed pieces.

In addition, to the field trial results, we compiled a generalised seed system model (S1 Table) showing the seed piece multiplication process with different multiplication cycles and timescales. The resulting amount of seed pieces per year and corresponding seed piece length is displayed in Fig 6. This allowed us to fully understand the impact of the reduction in seed piece size on existing seed multiplication protocols. Therefore, we first wanted to compare the different multiplication rates when using various seed piece lengths. The initial question was: “How long does it take with 8 cm seed pieces to produce the same amount as with the ‘traditional’ seed system of 5 years and 16 cm in Brazil or 24 cm in Uganda, respectively?”

The calculation results showed that a similar number of seed pieces can be obtained one year faster with 8 cm seed pieces (time difference = 364.95 days, equalling a time saving of 20.0%). We also compared the output for 24 cm seed pieces to the production speed of the 8 cm seed system: There, the time difference equals almost two years (= 679.33 days, Fig 6).

We assumed that the treatment technology is applied in each step where seed stems or shoot plantlets are used (but not in the initial tissue culture cycle), in combination to a reduction in their sizes (factor 2 or 3 –Brazil and Uganda, respectively). In addition, the model was based on data and multiplication factors obtained from Brazil (S1 Table). As this model is linear, the time savings are only dependent on the underlying multiplication factors.

## Discussion

The resilience and productivity of clonally propagated crops rely heavily on the availability of clean planting material and the use of improved genetics to limit the spread of seed-borne diseases [28]. However, apart from potato, there are relatively few established production and distribution systems for certified, disease-free planting material for clonal crops [12]. This is particularly important for cassava, in which seed-borne diseases can cause substantial losses of crop productivity [8]. As a response, several countries aim to install delivery channels that would supply farmers with certified, disease-free planting material [28]. However, formal seed systems for cassava are still rare, because of the crop’s low multiplication rate and the thus limited viability of seed multiplication. Our work aimed to develop solutions to increase the crop’s multiplication rates to make the production of planting material more efficient.

Numerous procedures already exist for rapid production of vegetatively propagated planting material. These include *in-vitro* techniques for clonal multiplication [29], micropropagation of meristem cultures [30, 31], as well as approaches based on two-node seed pieces which are planted under controlled conditions and require transplanting upon seedling establishment [32]. Thus, these methods tend to be costly both in terms of equipment and infrastructure, potentially preventing their large-scale use to provide farmers with planting material [33]. These methods are mostly limited to the initial multiplication cycles, involving varietal cleaning and basic planting material multiplication.

The approach described in the present paper enables increased multiplication efficiency not only under controlled conditions but also directly in the field, removing the need for green- and nethouses as well as transplanting. We were able to demonstrate the feasibility of shortening cassava seed pieces to 8 cm when using appropriate seed treatment formulations. This represents one-half to one-third of the conventionally used 16 or 24 cm pieces (Brazil and Uganda, respectively). This reduction is possible without negative effects on crop development or productivity. On the contrary: the proposed seed treatment solution has the potential to boost the plants’ early establishment and resilience to both biotic and abiotic stresses (Figs 3–5). The results thus show that the seed pieces can be reduced to one-third of conventional size without negatively affecting germination, crop vigour or productivity. As the multiplication rates increase, transport and storage bulk decreases, and smaller areas are needed for seed multiplication. At least one multiplication cycle (equivalent to one year) can be omitted from traditional seed multiplication protocols.

Interestingly, the initial vigour advantage lasts up to harvest, with plants derived from treated seed pieces performing better in terms of both above (28%) and below ground biomass (24% for fresh root yield and 26% for dry yield) than plants from untreated seed pieces. This has a dual benefit as both root and shoot yield can lead to sellable produce as shoot yield, in particular is also crucial for seed multiplication processes.

The treatment’s effect on increasing early plant vigour can most likely be traced to the systemic insecticidal constituent. The use of such compounds on seeds has been shown to provide advantages related to initial pest and disease control, as well as indirect effects resulting in enhanced plant vigour [17, 34, 35], as a result of the biosynthesis of specific functional proteins that help the plants to overcome several adverse environmental growing factors [36]. However, this requires careful choice of the agrochemicals because some active ingredients can cause serious phytotoxic effects (as observed in S1 Fig), and also reported in other crops [37].

Apart from increasing multiplication rates and providing a means for pest and disease control, seed treatment approaches that lead to a reduction of seed piece length can offer a number of additional advantages. These include easier logistics thanks to less bulky planting material and better options for crop planting. The latter is of particular importance, as mechanised equipment for planting normally requires small, uniform seed pieces. Furthermore, we believe that this technology can have beneficial impacts on crop management: by supporting row planting and an earlier closure of the canopy, weed control interventions could be reduced (Fig 4).

Lastly, apart from improving early plant vigour and resilience, systemic insecticides can offer systemic protection against pests. These include whiteflies, which–as well as causing significant feeding damage–are the most important vector of the two most prevalent cassava virus diseases in Africa [38]. Therefore, assessing a potential protective effect of such treatments might lead to the identification of additional benefits in areas with a high pest and vector prevalence [39]. However, further trials are needed to elucidate this potential systemic insecticidal effectiveness.

Overall, the technology described in the present study may hold considerable promise in facilitating the development of viable delivery channels for both clean planting material and improved genetics of cassava. However, there are additional factors that need to be considered when delivering this technology to cassava growers and further dialogue with relevant stakeholders is required to ensure the sustainable establishment of more formal seed multiplication and distribution systems.

We believe that there is farmer demand for planting material with disease control, germination and genetics in both Latin America and Africa. We would, therefore, suggest a paradigm shift for investment to support both crop improvement as well as seed systems development. The aim must be to improve farmer access to superior genetics and clean planting material, in order to reduce yield gaps.

The technology described in the present study has the potential to invigorate seed systems for clonal crops by making them more efficient. This could further help develop commercially viable and sustainable supply channels for quality planting material, making production systems for clonal crops more productive and resilient. This will be of particular importance to smallholder farmers, who rely heavily on clonal crops as a food and income source.

## Supporting information

S1 Fig Phytotoxicity symptoms of cassava seeds treated with different agrochemicals, evaluated 30 days after planting in a growth chamber. 8 cm seed pieces of the ‘Cascuda’ variety were treated with active ingredients of seed treatment formulations for sugarcane at dosages of 5% and 10% of original application rates, with two controls (Unt1 = cassava seeds treated only with water; Unt2 = cassava seeds were treated only with latex, 2%). ME+FL+AZ (fungicides: mefenoxam, fludioxonil, and azoxystrobin), TB (fungicide: thiabendazole), TM (insecticide: thiamethoxam). Error bars show standard errors. Different upper-case and lower-case letters indicate significant differences between the controls and treatments at 5% and 10% dosage, respectively (*p* < 0.05).
(TIF)

S2 Fig Growth performance of cassava seeds treated with different agrochemicals, evaluated 30 days after planting in a growth chamber. 8 cm seed pieces of the ‘Cascuda’ variety were treated with active ingredients of agrochemicals at 1.1%, 2.2% and 4.4% of the initial product formulation (as ‘Reference’ in Table 3), with one control (Unt1 = cassava seeds treated only with water). ME+FL (fungicides: mefenoxam and fludioxonil), ME+FL+TB (fungicides: mefenoxam fludioxonil and thiabendazole). Error bars show standard errors. Different upper-case, lower-case letters and numerals indicate significant differences between the controls and treatments at 1.1%, 2.2% and 4.4% dosage, respectively (*p* < 0.05).
(TIF)

S3 Fig Growth performance of cassava seeds in the root rot-infested substrate, evaluated 30 days after planting in a growth chamber. 8 cm seed pieces of the ‘Cascuda’ variety were treated with active ingredients of agrochemicals at dosages of 1.1%, 2.2% and 4.4% of the initial product formulation (as ‘Reference’ in Table 3), with one control (Unt1 = cassava seeds treated only with water). ME+FL (fungicides: mefenoxam and fludioxonil) and ME+FL+TB (fungicides: mefenoxam, fludioxonil, and thiabendazole). Error bars show standard errors. Different upper-case, lower-case letters and numerals indicate significant differences between the controls and treatments at 1.1%, 2.2% and 4.4% dosage, respectively (*p* < 0.05).
(TIF)

S1 Table Underlying multiplication factors of a generalised seed system. Multiplication factors are estimations from a Brazilian seed system model. Multiplication factors are indicated per step and cumulative, indicating the multiplication factor per cycle and the resulting additive amount of seed pieces after completion of the current including the previous cycles. Step duration is indicated in months. Propagation location as well as input and output material are specified for the corresponding step. After completion of all of the multiplication cycles, the produced seed material is assumed to be sold and used for commercial root production.
(DOCX)
