# Supplementary figures and images for: A novel seed treatment-based multiplication approach for cassava planting material
Source: PLoS One. 2020 Mar 6;15(3):e0229943. doi: 10.1371/journal.pone.0229943 (PMC7059944; doi:10.1371/journal.pone.0229943)

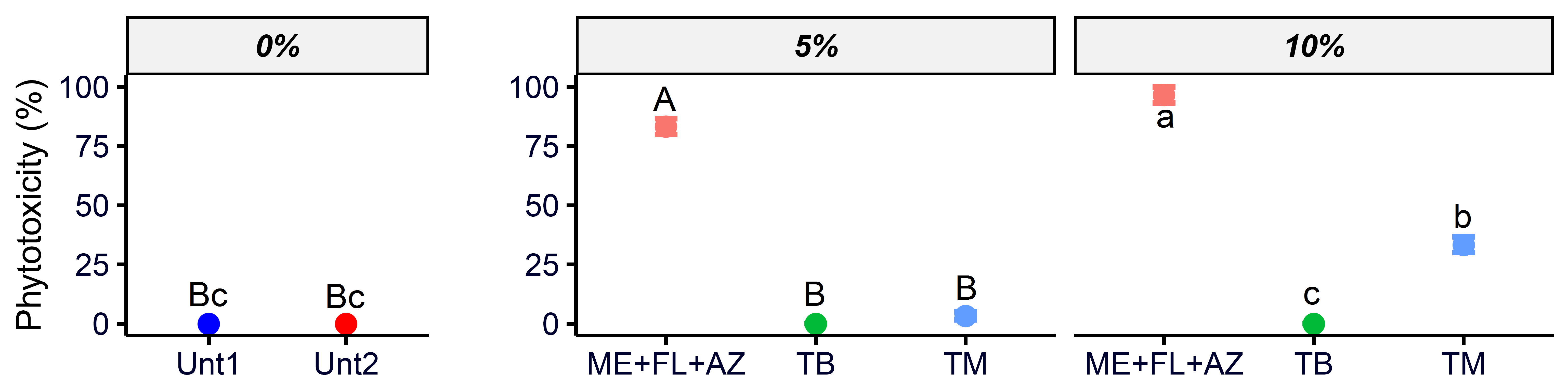

Supplement: S1 Fig — 8 cm seed pieces of the ‘Cascuda’ variety were treated with active ingredients of seed treatment formulations for sugarcane at dosages of 5% and 10% of original application rates, with two controls (Unt1 = cassava seeds treated only with water; Unt2 = cassava seeds were treated only with latex, 2%). ME+FL+AZ (fungicides: mefenoxam, fludioxonil, and azoxystrobin), TB (fungicide: thiabendazole), TM (insecticide: thiamethoxam). Error bars show standard errors. Different upper-case and lower-case letters indicate significant differences between the controls and treatments at 5% and 10% dosage, respectively (p < 0.05). (TIF) [file pone.0229943.s001.tif]

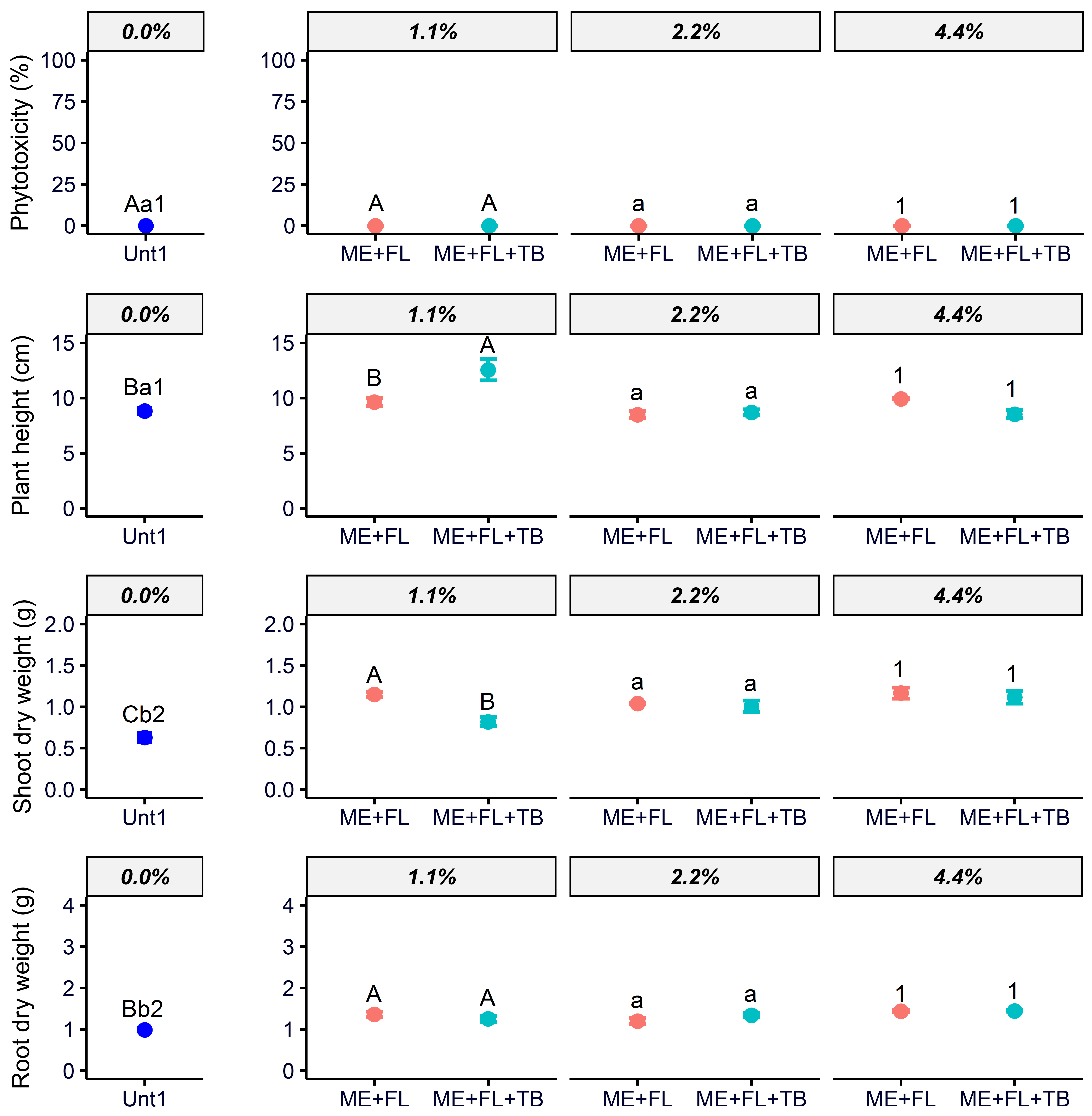

Supplement: S2 Fig — 8 cm seed pieces of the ‘Cascuda’ variety were treated with active ingredients of agrochemicals at 1.1%, 2.2% and 4.4% of the initial product formulation (as ‘Reference’ in Table 3), with one control (Unt1 = cassava seeds treated only with water). ME+FL (fungicides: mefenoxam and fludioxonil), ME+FL+TB (fungicides: mefenoxam fludioxonil and thiabendazole). Error bars show standard errors. Different upper-case, lower-case letters and numerals indicate significant differences between the controls and treatments at 1.1%, 2.2% and 4.4% dosage, respectively (p < 0.05). (TIF) [file pone.0229943.s002.tif]

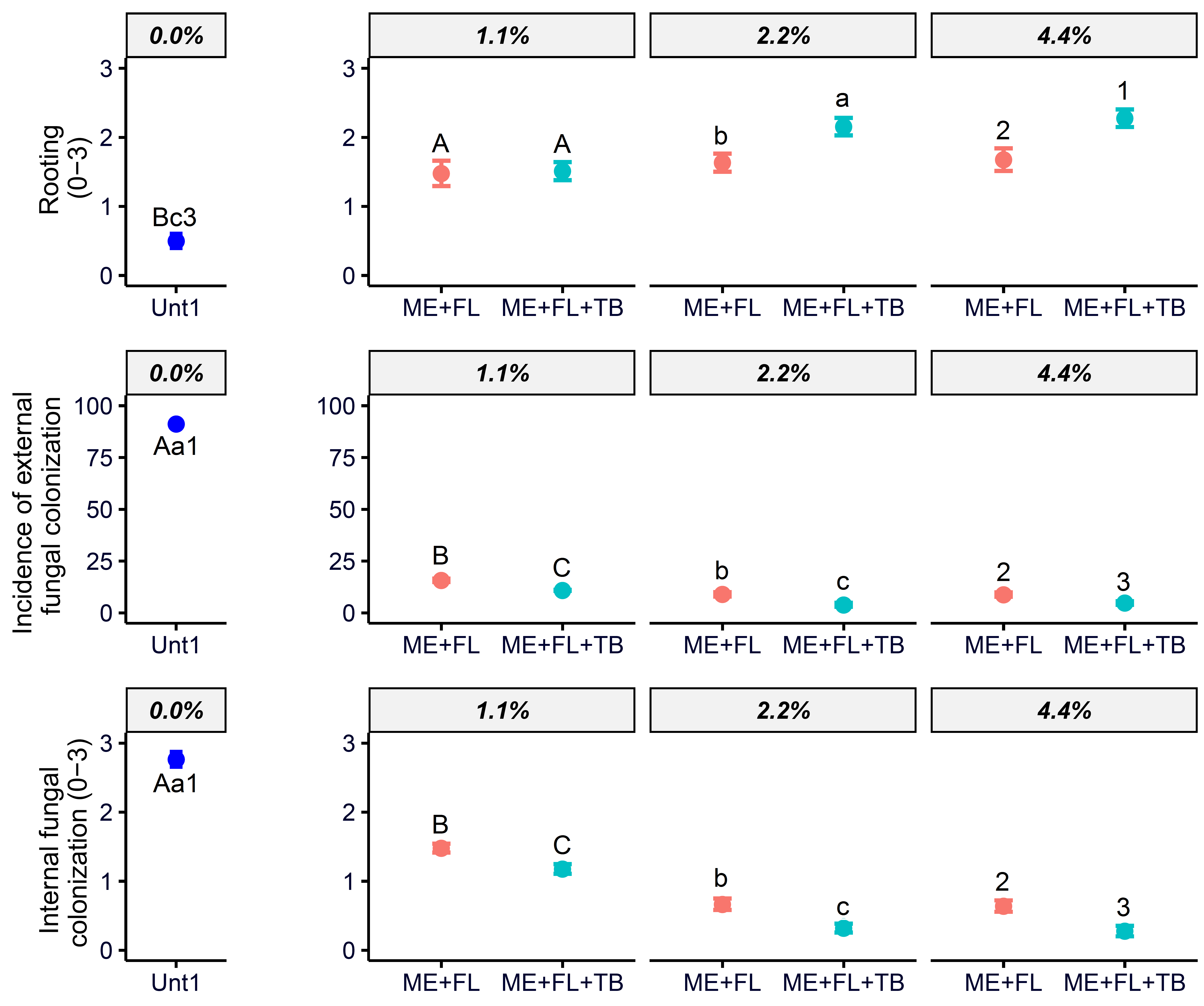

Supplement: S3 Fig — 8 cm seed pieces of the ‘Cascuda’ variety were treated with active ingredients of agrochemicals at dosages of 1.1%, 2.2% and 4.4% of the initial product formulation (as ‘Reference’ in Table 3), with one control (Unt1 = cassava seeds treated only with water). ME+FL (fungicides: mefenoxam and fludioxonil) and ME+FL+TB (fungicides: mefenoxam, fludioxonil, and thiabendazole). Error bars show standard errors. Different upper-case, lower-case letters and numerals indicate significant differences between the controls and treatments at 1.1%, 2.2% and 4.4% dosage, respectively (p < 0.05). (TIF) [file pone.0229943.s003.tif]
