# Supplementary material for: A novel seed treatment-based multiplication approach for cassava planting material
Source: PLoS One. 2020 Mar 6;15(3):e0229943. doi: 10.1371/journal.pone.0229943 (PMC7059944; doi:10.1371/journal.pone.0229943)
Supplement: S1 Table — Multiplication factors are estimations from a Brazilian seed system model. Multiplication factors are indicated per step and cumulative, indicating the multiplication factor per cycle and the resulting additive amount of seed pieces after completion of the current including the previous cycles. Step duration is indicated in months. Propagation location as well as input and output material are specified for the corresponding step. After completion of all of the multiplication cycles, the produced seed material is assumed to be sold and used for commercial root production. (DOCX) [file pone.0229943.s004.docx]

**Table S1**: Underlying multiplication factors of a generalised seed system. Multiplication factors are estimations from a Brazilian seed system model. Multiplication factors are indicated per step and cumulative, indicating the multiplication factor per cycle and the resulting additive amount of seed pieces after completion of the current including the previous cycles. Step duration is indicated in months. Propagation location as well as input and output material are specified for the corresponding step. After completion of all of the multiplication cycles, the produced seed material is assumed to be sold and used for commercial root production.

| **Step** | **Cycle** | **Propagation** | | | **Time (months)** | **Multiplication Factor (24cm)** | | **Multiplication Factor (16cm)** | | **Multiplication Factor (8cm)** | |
| --- | --- | --- | --- | --- | --- | --- | --- | --- | --- | --- | --- |
|  |  | In | Location | Out |  | Step | Cumulative | Step | Cumulative | Step | Cumulative |
| 1 Nuclear G1 | C0 | Cuttings | Tissue Culture Lab | Tissue Culture Plantlets | 6 | 1 | 1 | 1 | 1 | 1 | 1 |
| 2 Pre-Basic G2 | C1 | Tissue Culture Plantlets | Greenhouse multiplication | Shoot Plantlets | 6 | 9 ${= f}_{1 (24cm)}$ | 9 | 9${= f}_{1 (16cm)}$ | 9 | 9 ${= f}_{1 (8cm)}$ | 9 |
| 3 Basic G3 | C1 | Stems | Nursery Field | Stems | 12 (2*6) | 10.6 ${= f}_{2 (24cm)}$ | 95.4 | 16 ${= f}_{2 (16cm)}$ | 144 | 32 ${= f}_{2 (8cm)}$ | 288 |
| 4 Certified G4 | C1 | Stems | Nursery Field | Stems | 12 (2*6) | 10.6${= f}_{3 (24cm)}$ | 1’011.2 | 16${= f}_{3 (16cm)}$ | 2’304 | 32${= f}_{3 (8cm)}$ | 9’216 |
|  | C2 | Stems | Nursery Field | Stems | 12 | 5.3${= f}_{4 (24cm)}$ | 5’359.6 | 8${= f}_{4 (16cm)}$ | 18’432 | 16${= f}_{4 (8cm)}$ | 147’456 |
|  | C3 | Stems | Nursery Field | Stems | 12 | 5.3${= f}_{5 (24cm)}$ | 28’405.7 | 8${= f}_{5 (16cm)}$ | 147’456 | 16${= f}_{5 (8cm)}$ | 2’359’296 |
| Commercial Root Production |  |  |  |  |  |  |  |  |  |  |  |
